# Is Mandatory Prospective Trial Registration Working to Prevent Publication of Unregistered Trials and Selective Outcome Reporting? An Observational Study of Five Psychiatry Journals That Mandate Prospective Clinical Trial Registration

**DOI:** 10.1371/journal.pone.0133718

**Published:** 2015-08-19

**Authors:** Amelia Scott, Julia J. Rucklidge, Roger T. Mulder

**Affiliations:** 1 Department of Psychology, University of Canterbury, Christchurch, New Zealand; 2 Department of Psychological Medicine, University of Otago, Christchurch, New Zealand; Tilburg University, NETHERLANDS

## Abstract

**Objective:**

To address the bias occurring in the medical literature associated with selective outcome reporting, in 2005, the International Committee of Medical Journal Editors (ICMJE) introduced mandatory trial registration guidelines and member journals required prospective registration of trials prior to patient enrolment as a condition of publication. No research has examined whether these guidelines are impacting psychiatry publications. Our objectives were to determine the extent to which articles published in psychiatry journals adhering to ICMJE guidelines were correctly prospectively registered, whether there was evidence of selective outcome reporting and changes to participant numbers, and whether there was a relationship between registration status and source of funding.

**Materials and Methods:**

Any clinical trial (as defined by ICMJE) published between 1 January 2009 and 31 July 2013 in the top five psychiatry journals adhering to ICMJE guidelines (The American Journal of Psychiatry, Archives of General Psychiatry/JAMA Psychiatry, Biological Psychiatry, Journal of the American Academy of Child and Adolescent Psychiatry, and The Journal of Clinical Psychiatry) and conducted after July 2005 (or 2007 for two journals) was included. For each identified trial, where possible we extracted trial registration information, changes to POMs between publication and registry to assess selective outcome reporting, changes to participant numbers, and funding type.

**Results:**

Out of 3305 articles, 181 studies were identified as clinical trials requiring registration: 21 (11.6%) were deemed unregistered, 61 (33.7%) were retrospectively registered, 37 (20.4%) had unclear POMs either in the article or the registry and 2 (1.1%) were registered in an inaccessible trial registry. Only 60 (33.1%) studies were prospectively registered with clearly defined POMs; 17 of these 60 (28.3%) showed evidence of selective outcome reporting and 16 (26.7%) demonstrated a change in participant numbers of 20% or more; only 26 (14.4%) of the 181 the trials were prospectively registered and did not alter their POMs or the time frames at which they were measured. Prospective registration with no changes in POMs occurred more frequently with pharmaceutical funding.

**Discussion:**

Although standards are in place to improve prospective registration and transparency in clinical trials, less than 15% of psychiatry trials were prospectively registered with no changes in POMs. Most trials were either not prospectively registered, changed POMs or the timeframes at some point after registration or changed participant numbers. Authors, journal editors and reviewers need to further efforts to highlight the value of prospective trial registration.

## Introduction

In 2005, the International Committee of Medical Journal editors (ICMJE; www.icmje.org) introduced a policy [1] to require registration of all clinical trials into a registry prior to enrolment of participants. This policy was designed to address the increasing concern that negative trials were not being published [2], leading to a biased literature. Prospective declaration of primary outcomes prior to analysing data is also vital to ensure that analyses cannot influence which measures are reported on in the final publication. These problems often resulted in erroneous conclusions about efficacy, such that clinical guidelines were based on an incomplete and in some cases fraudulent literature [3–8]. To address these problems, the ICMJE developed trial registries as a public platform where information about trial protocols and pre-specified outcome measures for treatment trials could be made publicly available [9].

Journal members of ICMJE began requiring, as a condition of publication, evidence of prospective registration of any clinical trial [1]. The ICMJE define an acceptable registry as one which includes at a minimum a unique identifying number, statements of the intervention and comparison studied, the study hypothesis, definitions of primary and secondary outcome measures, eligibility criteria, key trial dates, target number of subjects, funding source, and contact information for the principal investigator [1]. However, research has highlighted that there are ongoing challenges with adherence to ICMJE’s registration policy, with studies showing continued publication of unregistered studies [10], even in journals that require mandatory registration [11–13]. While it appears that timely registration of trials is improving [13], about half of published trials are not being prospectively registered, with many clinical trials not being registered at all [12, 14, 15]. Selective outcome reporting and biased publication of positive findings is also evident across medical disciplines [12, 16–19], even after the adoption of the ICMJE guidelines.

To date, no study has investigated to what extent psychiatry has adopted ICMJE guidelines as a condition of publication and when they do require registration, whether this requirement is influencing the types of trials being published. Our study had four objectives: 1) to determine the prevalence of articles published in psychiatry journals adhering to ICMJE guidelines that were correctly prospectively registered since the implementation of the ICMJE policy; 2) of those trials identified as prospectively registered with clear POMs, to examine the consistency between POMs in the trial registries and published articles including whether there were retrospective changes to the POMs in the registry and to establish whether any discrepancies between the registries and published articles seemed to favor statistical significance; 3) in prospectively registered trials with clear POMs, to examine consistencies between the participant numbers listed in the trial registries and the published articles; and 4) to determine for clinical trials identified, whether source of funding was related to better adherence with ICMJE guidelines.

## Methods

### Selection of Journals

We focused on the top impact factor (IF) rated journals in psychiatry that also required as a condition of publication, prospective trial registration of clinical trials, as we expected that these journals would be more likely to adhere to the ICMJE guidelines. At the time we began our study in January 2013, *The American Journal of Psychiatry* (AJP), *Archives of General Psychiatry/JAMA Psychiatry*, *Biological Psychiatry* (BP), *Journal of the American Academy of Child and Adolescent Psychiatry* (JAACAP), and *The Journal of Clinical Psychiatry* (JCP) were identified as the top Impact journals in psychiatry that all required prospective registration of trials as a condition of publication based on their Instructions for Authors.

### Search/ Eligibility/Selection of Articles

We included all articles published in the selected journals between 1 January 2009 and 31 July 2013 in our screening process. From these five journals within this time frame, 3,305 articles were identified. Author AS determined which articles were within the scope of the study, first judged by reading the title and abstract and then by reading the full text of the remaining potentially relevant articles.

The ICMJE initiated its trial registration guidelines on 1 July 2005. While *JCP*, *AJP* and *JAMA Psychiatry* follow the ICMJE guidelines of prospective registration for anything that began patient enrolment on or after 1 July 2005, *BP and JAACAP* require prospective registration for trials that began patient enrolment after 1 February 2007. We used the date stipulated by the journal rather than the ICMJE date to determine which clinical trials required prospective registration, ensuring that there was no ambiguity in whether a trial was expected to be prospectively registered by the journal editor.

For the purpose of registration, the ICMJE defines a clinical trial as ‘any research project that prospectively assigns human subjects to intervention or comparison groups to study the cause-and-effect relationship between a medical intervention and a health outcome’ [1]. All of the included journals, except for *JCP* (which refers to the World Health Organisation definition (http://www.who.int/ictrp/glossary/en/)), refer directly to this statement in their clinical trial registration requirements. We determined studies to be treatment trials if they: 1) were evaluating a group of humans who were not identified as ‘healthy participants’; 2) were administering a treatment to this group of people; and 3) were giving the treatment with the intention of improving a health-related outcome. Broadly, we interpreted a health-related outcome as a psychological or psychiatric outcome variable. Therefore, we deemed that changes in fMRI or other biomarkers did not meet the criteria of a health-related outcome. Commentary studies, follow-up studies, secondary publications or secondary analyses of results (identified by reading the full article to identify if there was any mention of a previous publication and checking with the trial registry or authors when necessary), and discussion articles were excluded from our analysis. All prospective designs (e.g., randomized controlled trials (RCTs) and open-label trials) were included as long as it was deemed the study met the three criteria. In cases where this was unclear, the three authors considered the study, with disagreements resolved by consensus and in some cases, by emailing the corresponding author of the study. We took a conservative approach in that if there was uncertainty, we erred on the side of the trial not requiring registration.

In order to undertake our analysis of discrepancies, we required full access to trial protocols. While the ICMJE guidelines states that trials must be registered in registries that are freely and readily available, some trial protocols were published in registries that did not meet these criteria. Therefore, these trials were not able to be included in our in depth analysis of discrepancies as we will not be able to access their registry information.

### Identification of Registration

Each published trial was thoroughly examined to determine if the trial was registered according to a modified version of Mathieu et al’s [12] methods as follows. First, each article was searched to see if the author(s) had reported the trial registration number in the text. If the trial registration details could not be found this way, the most common trial registries were searched using the treatment and condition being treated as search parameters. Trial registries searched at this stage were: ClinicalTrials.gov, International Standard Randomized Controlled Trial Number Register (www.controlled-trials.com), and the registry of the country of the first or corresponding author (e.g., Australian New Zealand Clinical Trials Registry (www.anzctr.org.au) and Nederlands Trial Register (www.trialregister.nl)). Search results were confirmed as a match by comparing the description of the trial and identifying the responsible author for the trial registration as one of the authors of the article. If no trial registry was found in this search, author JR emailed the corresponding author to ask if the trial was registered and, if so, what the trial identification number was. If there was no response, a follow-up email was sent again asking for registration details. If no response was received after this process, the article was determined to be unregistered.

### Data Extraction

The start date for each study was taken primarily from the text of the publication. If no start date was included in the article, the date stipulated in the trial registry was used. In the instance of a discrepancy, the start date in the article was used as we judged that this date would be more accurate. The date of trial registration was extracted from the registry in order to evaluate whether the trial was prospectively registered. For retrospectively registered trials, we assessed how long after the study start date the registration occurred.

Further data extracted included the title of the journal and article, name(s) of author(s) and the POM(s) listed in the article and ‘current primary outcome’ section of the trial registry. Data was extracted and documented by author AS.

For the analysis of participant numbers, we extracted the number of participants from the article. We used the ‘number randomized’ or in non-randomized studies, the number of participants included at baseline with intention to treat before any dropouts. For the trial registry numbers, we tracked changes in the protocols where possible so as to use the original number of participants entered when the trial protocol was first registered. Clinicaltrials.gov allows the public to track changes easily; however, this is not a feature of all trial registers. For protocols published in registries that do not allow changes to be tracked, such as ISRCTN, we simply used the target number of participants listed in the protocol.

Funding information from the trial registry was extracted to document the relationship between registration status, discrepancies and funder. This was done by first searching the trial registry, then in cases where more information was required or where the trial was unregistered, the funding information identified in the article was used. Funders were classified as either public (e.g. universities, hospitals), private (e.g. Mayo Clinic), or funded by a pharmaceutical industry (e.g. GlaxoSmithKline). Hospitals and universities were classified as public funding sources.

### Identification of Discrepancies

Once articles were determined to be relevant to our study, we examined whether we were able to compare the primary outcome measures in the article to those found in the trial protocols. We first established whether the POMs and their listed timeframes were clearly defined in the article, and later, once trial protocols were located, in the trial registry. An unclear POM was one that did not list a specific measure, for example: ‘Change in depression severity’ as opposed to listing a specific measure such as the Hamilton Depression Rating Scale. Articles that had no primary outcome or an unclear primary outcome measure in the article or trial registry were not able to be analysed in our in depth analysis of discrepancies as we could not establish whether a change in POM had occurred.

The remaining prospectively registered articles were evaluated for their consistency between the POMs and participant numbers listed in the article and the trial registry. Outcome measure discrepancies were defined according to Mathieu et al.’s [12] classification system as follows: 1) The registered primary outcome was reported as a secondary outcome in the published article, 2) the registered primary outcome was omitted in the published report, 3) a new primary outcome was introduced in the published article (ie, a registered secondary outcome that becomes primary in the article or an outcome that does not appear at all in the registry but is introduced as primary in the article), 4) the published primary outcome was described as a secondary outcome in the registry, and/or 5) the timing of assessment of the registered and published primary outcomes differed.

Articles where discrepancies were found based on Mathieu et al.’s system were assessed to see if the discrepancies favored statistically significant results. All discrepancies were independently classified by all authors and any differences discussed and resolved by consensus. As with Mathieu et al.’s [12] and Chan et al.’s [6] studies, a discrepancy was considered to favor statistically significant results if a new statistically significant efficacy primary outcome was introduced, or when a registered primary outcome was omitted or defined as non-primary in the published article.

Analysis of discrepancies was first based on the difference between the current primary outcome(s) in the trial registry and article. However, as trial registries allow authors to update the information at any time after registration, including the primary outcomes, even after the study is completed, we also examined whether the POMs in the trial registries were updated *after the trial was registered*. In such a situation, there would be a match between *current* POMs in the registry and the article, but a mismatch between *original* POMs and the article. The registry tracks the date changes were made to POMs such that we could also assess whether changes to POMs in the registry were made before or after submission of the article to the journal. As we were interested in whether researchers made a substantial change to the POMs, potentially resulting in selective outcome reporting, we only identified a change when the ‘original primary outcome measure’ section of the registry listed different outcome measure(s) or timeframe(s) to the ‘current primary outcome measure’ section. Cases where additional clarifying information about existing outcomes was added but no change was made to the POM were not identified as having a discrepancy.

All unregistered, retrospectively registered and properly registered articles with clear POMs were checked independently by the third author (RM, a psychiatrist) to verify classification decisions made by the first two authors. All inconsistencies were discussed and a consensus decision made, always making conservative decisions with any disagreement.

## Results

**Fig 1:**
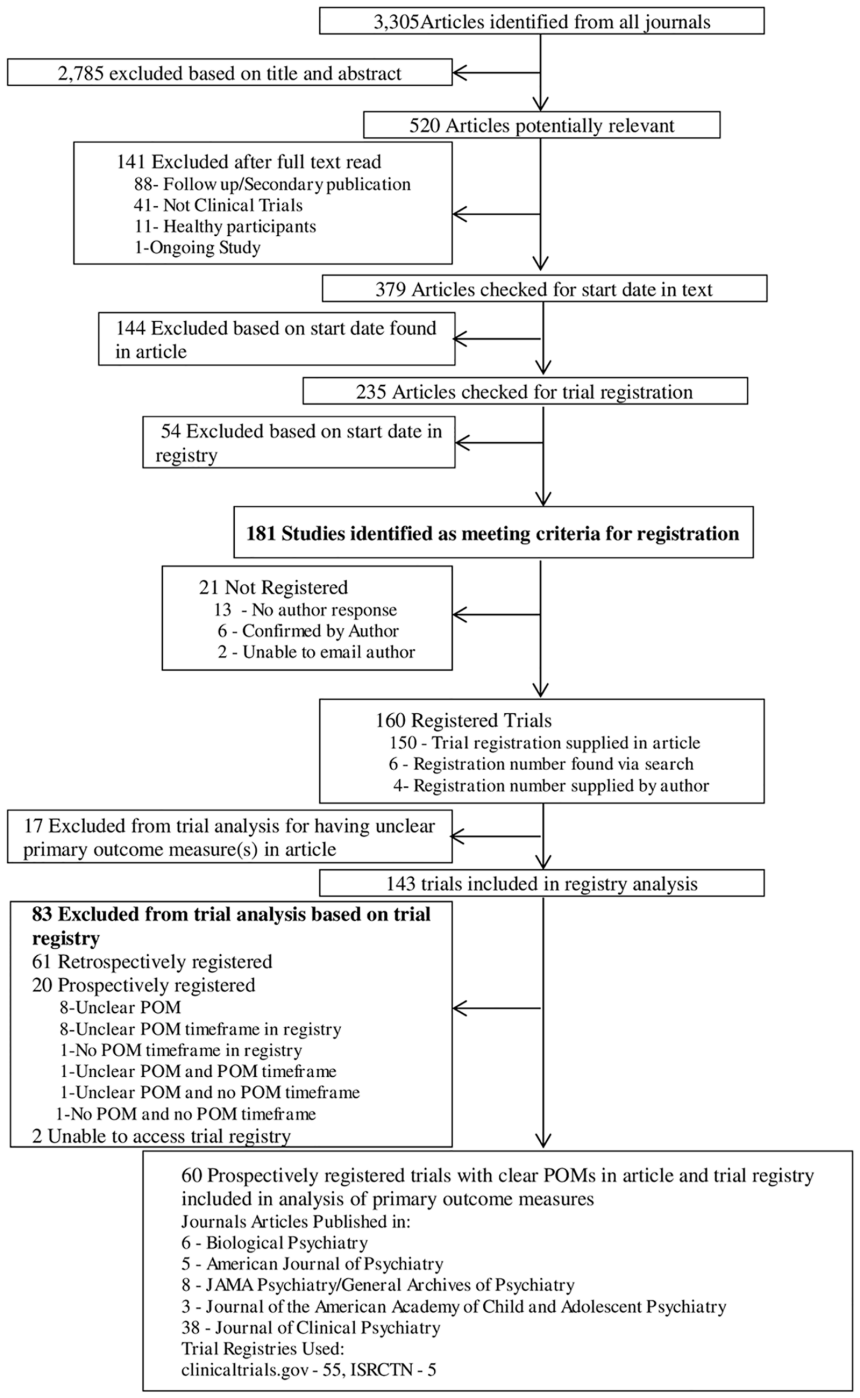
Flowchart of Articles Used to Assess Registration and Selective Reporting.

From 3,305 articles identified from all five journals spanning the time frame of January 1^st^ 2009 to July 31^st^ 2013, 520 articles were found to be potentially relevant to our study based on the title and abstract (Fig 1). Upon further inspection, 141 articles were excluded due to being outside of the scope. Of the 379 remaining articles, 198 reported having begun patient enrolment before 1 July 2005 (or 1 February 2007 for those articles published in *JAACAP* and *BP*). Out of the remaining 181 studies determined to require prospective trial registration, 21 (11.6%) were deemed to be unregistered. Of the 160 registered trials, 17 had an unclear primary outcome measure(s) in the article, 61 had been retrospectively registered, 20 had an unclear or imprecise POM and assessment times in the registry, and two had protocols in registries that were not freely available. Thirty (49.2%) of the 61 retrospectively registered trials took a year or more to be listed on the registry website (Table 1).

**Table 1:** Length of time taken after start date for trial registration to be created for trials that were retrospectively registered.

Length of time after start date that trial was registered	Number of Articles (%)
Less than 1 month after	7 (11.5)
1 month to less than 6 months after	14 (23)
6 months to less than 12 months after	10 (16.4)
1 year to less than 5 years after	28 (45.9)
5 years or more after	2 (3.3)

Of the 181 trials identified as requiring prospective registration, 60 (33.1%) were correctly registered before the onset of patient enrolment, with clear descriptions of POMs and the times participants would be assessed.

Among the 60 correctly registered articles (S1 File), 17 (28.3%) showed discrepancies between the outcome measures in the trial registry and the published article (Table 2) with the most commonly identified discrepancy being an omission in the published article of a declared POM in the registry. For the 17 articles with discrepancies, we were unable to determine the reason for the discrepancy for five because the POMs had been omitted from the article. For the remaining twelve, seven studies were considered as having made changes to favour statistically significant results and five were not.

**Table 2:** Differences in Primary Outcome Measures between Trial Registry and Published Article for Prospectively Registered Trials with Clear Descriptions of Outcome Measures and Differences in Primary Outcomes Favouring Statistically Significant Results.

	Number of Articles (%)
**Articles with primary outcome measure discrepancies between the trial registry and published article**	17^a,b^
Registered primary outcome(s) not mentioned in published article	9 (52.9)
New primary outcome(s) not in registry introduced in article	3 (17.7)
Different timing of assessment for primary outcome measure(s)	3 (17.7)
Primary outcome in article reported as secondary outcome in registry	3 (17.7)
Primary outcome in registry reported as secondary outcome in article	5 (29.4)
**Discrepancies in primary outcome reporting favouring statistically significant results**	
Yes	7 (41.2)
No	5 (29.4)
Not possible to determine	5 (29.4)

Forty-three (71.7%) studies were found to have no discrepancies between the article and the ‘current primary outcome measure’ section in the registry. However, as trial registries are able to be updated at any time by authors, and some clinical trial registries keep a record of updates, we also investigated whether changes had been made to the pre-specified POMs in the trial protocol. While three protocols were published in registries that do not track changes, we found that 17 of these 43 articles had retrospectively updated the POMs in their protocol after the start date of the trial by either adding, removing or altering an outcome measure or timeframe for a POM. One of these articles made these changes after the article had already been submitted for publication. In other words, of the 60 articles found to be correctly prospectively registered, only 26 of them had no evidence of discrepancies between the trial protocol and article and showed no retrospective changes to the POMs in the registry.

The differences in participant numbers between the article and the original entry in the trial registry ranged from no difference, to 67.9% change. Eight (13.3%) of the articles showed no change in participant numbers between the trial registry and the article. Of the articles displaying a difference in participant numbers between the original registry entry and the article, 30 (57.7%) of articles actually reported an increase in participants rather than a decrease. Thirty-two (53.3%) of the final 60 articles had a participant difference less than 20%, while a further 16 (26.7%) had a change in enrolment of between 20% and 50%. Changes of 50% or more occurred in four (6.7%) studies, with two studies having increased from their original target in the trial registry and two having decreased. Five of the articles’ trial protocols were published in the register ISRCTN which does not allow changes to be tracked. We were therefore unable to determine whether the participant number listed in the registry was updated retrospectively. In three cases, the participant number was only entered into the trial registry retrospectively.

Table 3 shows sources of funding as they pertain to the type of registration. Thirty-seven per cent of pharmaceutically-funded trials were properly registered with no changes to POMs versus 6% of private trials, 10.3% of publicly funded trials and 10% of those with a combination of funding. A chi square analysis of these percentages showed a significant group difference (χ2 (3) = 10.19, p < .05), indicating that there were more trials properly registered by the pharmaceutical companies than the private and publicly funded trials. This finding suggests that prospective registration of trials with no changes to POMs was more likely to occur with pharmaceutically-funded studies.

**Table 3:** Funding source of research across different categories of registration.

	Funded by pharmaceutical company (e.g. Bristol Myers Squibb)	Privately funded (e.g. Mayo Clinic)	Publicly funded (e.g. NIMH)	Combined funding
21 Unregistered articles^a^	3	6	8	3
61 Retrospectively registered articles	12	6	39	4
17 Articles with discrepancies between 'current POMs' in trial registry and article	2	3	11	1
17 Articles marked 'discrepancy free' than had retrospective updates to POMs in trial registry	12	1	3	1
26 Articles correctly registered with no POM discrepancies^b^	17	1	7	1

^a^one study reported no source of funding,

^b^includes 3 articles where we were unable to determine whether POMs retro updated in trial registry.

## Discussion

Based on a 4.5 year review of all studies published in the top five impact factor journals in psychiatry that also require prospective registration of clinical trials as a condition of publication, we found that of the 181 articles that were deemed to require registration, 21 (11.6%) were determined to be unregistered and 61 (33.7%) were retrospectively registered. A further 20 (11%), although prospectively registered, POMs were not clearly delineated in the registry. Only 60 (33.1%) articles were correctly prospectively registered with clear descriptions of the POMs in the article and trial registries but, *over half of these trials* made changes to their originally declared POMs. Indeed, only 26 (14.4%) of the 181 articles published were prospectively registered with no retrospective changes to POMs.

Therefore, even though guidelines of all the journals we investigated clearly state that trials started since 1 July 2005 (or 2007 for two of the journals) must have been *prospectively registered* in order to be published, almost half of trials requiring registrations did not meet this basic requirement, confirming a continued problem of researchers (and journals) fully observing the ICMJE guidelines [12–14, 20]. It also suggests that journal editors are either not checking trial registries for appropriate registration or determined that there were valid reasons for why the trial was not properly registered and chose to publish it. Communication with the editor of one of the journals (*AJP*) suggested that this latter reason was indeed the case.

We also determined that selective outcome reporting continues to be a problem, consistent with other research [18, 21–23], even with prospectively registered trials. Seventeen (28.3%) of the 60 correctly registered articles displayed evidence of selective outcome reporting. In at least seven of these cases, changes favored statistically significant findings; in another five, there was no evidence of changes favouring statistically significant findings; and in the remaining five, we had insufficient information to make a decision because the outcomes were not included in the publications. We did not assume that dropping a POM meant that it had been dropped because it was non-significant, this is simply one possibility for why a change like this may have occurred. Despite the extensive publications highlighting this significant problem for over two decades [8, 20], some researchers continue to be selective in their reporting or change a priori decisions (possibly based on findings), despite the knowledge that this practice can have significant repercussions for health decisions and policies and clinical guidelines [21]. If pre-specified POMs are excluded from published trial reports because the findings do not support the hypotheses, there is the potential to overestimate the effectiveness of a treatment or intervention resulting in a bias in the literature. As demonstrated by case studies on paroxetine [9], burying primary outcome data because it is not statistically significant can have dangerous implications for the individuals who eventually may end up using the treatment. Changing time frames can result in a drug appearing more effective as compared with placebo, as evidenced by the reporting of Ballenger et al [24] on Alprazolam. In this study, the researchers focused on the 4 week data despite the fact that the trial was 8 weeks. While at 4 weeks there was clear advantage of the drug over placebo, this advantage disappeared at the 8 week time point. If there is compliance among editors and authors, mandatory trial registration would constitute one step of ensuring an unbiased and open psychiatric literature. Other steps to ensure full transparency would include release of raw data sets to independent investigators and uploading into public databases the trial protocols.

Even in publications that appeared to show consistency between the registry and the publication, evidence of potential post-hoc changing of POMs in the registry was revealed. This means that POMs listed in the registries are *currently* consistent with the POMs reported in the articles; however, when one tracks the changes being made to the registry, original POMs had been altered in 17 of the 43 correctly registered articles at some point either during the course of the study or after the study was completed. Except for one trial where changes to the POMs were made after submission of the article (based on submission date and dates when changes were made to the registry), we could not determine if these changes were being made before or after statistical analyses and why the changes had been made. Even though the trial registries are meant to be transparent such that changes can be traced, they can be challenging to navigate and no matter how transparent the trial registries become [25], researchers should make clear in the publication why changes were made to POMs retrospectively. There could be benign reasons for changing POMs such as a better POM was found during the course of the trial or an error had been made.

Fifty two (86.7%) of the studies in our final sample of 60 prospectively registered studies with clearly identified POMs showed differences in participant enrolment between their first trial registry entry and the published article. However, 30 (50%) of these studies actually increased participant numbers from their first estimate in the registry. Of the 22 (36.7%) studies that had sample sizes smaller than the expected numbers listed in the trial registry, 12 (20%) were found to have a difference between 20% and 50% while a further two (3.3%) displayed a difference of 50% or more. The remaining eight studies had a difference in participant numbers less than 20%, a number expected if one considers dropouts. This change in sample size is better than that observed in other studies whereby the original sample size declared in the registry was achieved in about 60% of published papers [23]. While some authors did state the reasons for these differences, citing difficulties with recruitment, lack of funding and strict inclusion/exclusion criteria among other reasons for not reaching their target number of participants, it is also possible that in some cases these high levels of change may reflect early analysis to detect group differences.

In order to determine the original sample size and POMs listed in the trial protocols from when they were first created, we tracked the changes in the registry itself. This is a feature that is possible in registries such as Clinicaltrials.gov and ANZCTR, but not ISRCTN. In order to achieve full transparency of research, it is important to have information such as the dates and changes made to POMs and participant numbers in trial protocols freely and readily available.

We identified that it was not always obvious, to us or to study authors, whether registration was required for particular studies. For example, for some unregistered trials, while the authors did report on health related outcomes, these outcomes were not the primary focus of the paper and therefore, it is possible that authors were unclear as to whether registration was required. For trials that are not RCTs, researchers may not consider or be aware that nonRCTs would be classified as a clinical trial based on the ICMJE guidelines as they prospectively assigns human subject to an intervention in order to study the cause-and-effect relationship between the intervention and a health outcome. It is also possible that authors are not registering their studies or doing so retrospectively because they were unaware of the need for trial registrations. It takes time for the guidelines as well as the rationale behind the guidelines to come to the attention of researchers and some may simply have been unaware of the inception of clinical trial registries. It is also possible that the trial registries can be slow to register trials and while a trial may have been submitted prospectively, delays may have resulted in patients being recruited before final registration was granted. This may explain a time lag of a month, but is harder to justify for those where a year or more has passed.

Our study aimed to investigate those studies that were reporting the primary outcome measures of a clinical trial. It is not always easy to determine if a manuscript is reporting secondary outcomes or other areas of further investigation beyond the primary measures. In order to achieve this, we read each article in full to identify that it was not a secondary analysis and where necessary, checking trial registries. While we attempted to ensure the studies we included were the first report on the dataset, we acknowledge that it is possible that we may not have been able to identify every instance of secondary publications of results through this method. We are also aware that in some cases, it is acceptable for multiple reports to be published on a single trial, each reporting on different primary outcome measures. In these instances, it is possible that we may have identified a discrepancy in POMs between the article and the registry, whereas in fact, those POMs were analysed in a subsequent publication on the same study. In these instances, we advocate that any publication reporting on some but not all POMs for a trial should specifically outline that remaining POMs will be discussed in following publications.

The inception of trial registries is a step in the right direction to address a long standing challenge in psychiatry as well as other medical disciplines, of negative trials not being published as well as outcome measures being changed, added or deleted, depending on the results, creating a biased literature that cannot be relied on to adequately guide clinical decisions. Most journals do not mandate trial registration as a condition of publication and therefore it is to their credit that the journals we investigated have done so. Indeed, based on the journals we found, European and Australasian journals have been less likely to adopt mandatory registration. It takes time for guidelines to filter down to the researchers and this may be why so many studies are not prospectively registering trials nor appreciating the significance of doing so. It appears that researchers conducting trials funded by the pharmaceutical industry are more aware of the importance of properly registering trials and not making retrospective changes to POMs. However, the journals that are members of the ICMJE also hold a responsibility to ensure those guidelines are met, and if not, and they choose to publish the work, that it is clear to readers that they have deviated from the expectations stated in their guidelines to authors. While the journals may have good reasons for doing so, readers assume that the trials published in these journals are prospectively registered and therefore the journals have an obligation to make decisions that err from this standard fully transparent.

When new guidelines come along, it can take time for behavior to change. Therefore, maybe it should not be surprising how few researchers are correctly registering their trials. Journals are in a powerful position to change the behaviors of researchers simply by mandating they meet specific requirements before a paper will be published. Those journals that aspired to meet the ICMJE guidelines have clearly felt justified to deviate from them. Journal editors clearly allow some discretion when submissions are made; however, these editorial decisions are not currently included in the published manuscript. There is also a long way to go in terms of increasing the number of journals that deem prospective registration of clinical trials as sufficiently important to make it a requirement of publication.

Looking forward, the situation could be improved if some of the following suggestions were to be adopted: 1) member journals of the ICMJE should have a dedicated person checking trial registries, trials should simply not be published if they haven’t been prospectively registered as determined by the ICMJE or the journals should state clearly and transparently reasons why studies might be published without adhering to ICMJE guidelines, 2) if authors do change POMs or participant numbers or retrospectively register their trials, the reasons should be clearly outlined in the methods, 3) to further improve transparency, authors could upload the full clinical trial protocol, including all amendments, to the registry website and provide the raw data from a clinical trial in a format accessible to the research community, 4) greater effort needs to be made to ensure authors are aware of the importance of prospectively registering trials, by improving guidelines for submission [26] and when applying for ethical approval, and 5) finally, reviewers should not make decisions about the acceptability of a study for publication based on whether the findings are positive or negative as this may be implicitly encouraging authors to be selective in reporting results. Indeed, a recent study showed that only one third of peer reviewers examined registered trial information and reported any discrepancies to the journal editors [27]. It is clear that while there are guidelines in place to decontaminate the medical literature, authors, reviewers and journal editors need to work together to ensure that these guidelines are upheld.

## Supporting Information

S1 File Trials used in final analysis of selective outcome reporting. (DOCX)

S2 File Raw data files. (XLSX)
