# Supplementary material for: Is Mandatory Prospective Trial Registration Working to Prevent Publication of Unregistered Trials and Selective Outcome Reporting? An Observational Study of Five Psychiatry Journals That Mandate Prospective Clinical Trial Registration
Source: PLoS One. 2015 Aug 19;10(8):e0133718. doi: 10.1371/journal.pone.0133718 (PMC4546113; doi:10.1371/journal.pone.0133718)
Supplement: S1 File — (DOCX) [file pone.0133718.s001.docx]

S1

1. aan het Rot M, Collins KA, Murrough JW, Perez AM, Reich DL, et al. (2010) Safety and Efficacy of Repeated-Dose Intravenous Ketamine for Treatment-Resistant Depression. Biol Psychiatry: 67(2) 139-145.
2. Adler AL, Dirks B, Deas PF, Raychaudhuri A, Dauphin MR et al. (2013) Lisdexamfetamine Dimesylate in Adults With Attention-Deficit/ Hyperactivity Disorder Who Report Clinically Significant Impairment in Executive Function: Results From a Randomized, Double-Blind, Placebo-Controlled Study. J Clin Psychiatry: 74(7) 694-702.
3. Allen MH, Feifel D, Lesem MD, Zimbroff DL, Ross R et al. (2011) Efficacy and Safety of Loxapine for Inhalation in the Treatment of Agitation in Patients With Schizophrenia: A Randomized, Double-Blind, Placebo-Controlled Trial. J Clin Psychiatry: 72(10) 1313-21.
4. Bain EE, Apostol G, Sangal RB, Robieson WZ, McNeill DL et al. (2012) A Randomized Pilot Study of the Efficacy and Safety of ABT-089, a Novel α4β2 Neuronal Nicotinic Receptor Agonist, in Adults With Attention-Deficit/Hyperactivity Disorder. J Clin Psychiatry: 73(6) 783-9.
5. Bloch MH, Panza KE, Grant JE, Pittenger C and Leckman JF. (2013) N-Acetylcysteine in the Treatment of Pediatric Trichotillomania: A Randomized, Double-Blind, Placebo-Controlled Add-On Trial. J Am Acad Child Adolesc Psychiatry: 52(3) 231-40.
6. Brams M, Weisler R, Findling RL, Gasior M, Hamdani M et al. (2012) Maintenance of Efficacy of Lisdexamfetamine Dimesylate in Adults With Attention-Deficit/Hyperactivity Disorder: Randomized Withdrawal Design. J Clin Psychiatry: 73(7) 977-83.
7. Brecht S, Desaiah D, Marechal ES, Santini AM, Podhorna J et al. (2011) Efficacy and Safety of Duloxetine 60 mg and 120 mg Daily in Patients Hospitalized for Severe Depression: A Double-Blind Randomized Trial. J Clin Psychiatry: 72(8) 1086-94.
8. Brecht S, Desaiah D, Marechal ES, Santini AM, Podhorna J et al. (2013) Efficacy and Safety of Levomilnacipran Sustained Release 40 mg, 80 mg, or 120 mg in Major Depressive Disorder: A Phase 3, Randomized, Double-Blind, Placebo-Controlled Study. J Clin Psychiatry: 74(3) 242-8.
9. Buchanan RW, Keefe RSE, Lieberman JA, Barch DM, Csernansky JG et al. (2011) A Randomized Clinical Trial of MK-0777 for the Treatment of Cognitive Impairments in People with Schizophrenia. Biol Psychiatry: 69(5) 442-9.
10. Craske MG, Stein MB, Sullivan G, Sherbourne C, Bystritsky A et al. (2011) Disorder-Specific Impact of Coordinated Anxiety Learning and Management Treatment for Anxiety Disorders in Primary Care. Arch Gen Psychiatry: 68(4) 378-88.
11. Cusin C, Iovieno N, Iosifescu DV, Nierenberg AA, Fava M. (2013) A Randomized, Double-Blind, Placebo-Controlled Trial of Pramipexole Augmentation in Treatment-Resistant Major Depressive Disorder. J Clin Psychiatry: 75(7) e636-41
12. Feifel D, Macdonald K, Nguyen A, Cobb P, Warlan H et al. (2010) Adjunctive Intranasal Oxytocin Reduces Symptoms in Schizophrenia Patients. Biol Psychiatry: 68(7) 678-80.
13. Grant PM, Huh GA, Perivoliotis D, Stolar NM and Beck AT (2012) Randomized Trial to Evaluate the Efficacy of Cognitive Therapy for Low-Functioning Patients With Schizophrenia. Arch Gen Psychiatry: 69(2) 121-7.
14. Gray KM, Carpenter MJ, Baker NL, DeSantis SM, Kryway E (2012) A Double-Blind Randomized Controlled Trial of N-Acetylcysteine in Cannabis-Dependent Adolescents. AM J Psychiatry: 169(8) 805-12.
15. Hardan AY, Fung LK, Libove RA, Obukhanych TV, Nair S (2012) A Randomized Controlled Pilot Trial of Oral N-Acetylcysteine in Children with Autism. Biol Psychiatry: 71(11) 956-61.
16. Hellerstein DJ, Stewart JW, McGrath PJ, Deliyannides DA, Batchelder ST et al. (2012) A Randomized Controlled Trial of Duloxetine Versus Placebo in the Treatment of Nonmajor Chronic Depression. J Clin Psychiatry: 73(7) 984-91.
17. Henigsberg N, Mahableshwarkar AR, Jacobsen P, Chen Y and Thase ME (2012) A Randomized, Double-Blind, Placebo-Controlled 8-Week Trial of the Efficacy and Tolerability of Multiple Doses of Lu AA21004 in Adults With Major Depressive Disorder. J Clin Psychiatry: 73(7) 953-9.
18. Herring WJ, Wilens TE, Adler LA, Baranak C, Liu K et al. (2012) Randomized Controlled Study of the Histamine H3 Inverse Agonist MK-0249 in Adult Attention-Deficit/Hyperactivity Disorder. J Clin Psychiatry: 73(7) e891-8.
19. Hoffmann VP, Case M and Jacobson JG (2012) Assessment of Treatment Algorithms Including Amantadine, Metformin, and Zonisamide for the Prevention of Weight Gain With Olanzapine: A Randomized Controlled Open-Label Study. J Clin Psychiatry: 73(2) 216-23.
20. Holtzheimer PE, Kelley ME, Gross RE, Filkowski MM, Garlow SJ et al. (2012) Subcallosal Cingulate Deep Brain Stimulation for Treatment-Resistant Unipolar and Bipolar Depression. Arch Gen Psychiatry: 69(2) 150-8.
21. Houston JP, Tohen M, Degenhardt EK, Jamal HH, Liu LLL et al. (2009) Olanzapine-Divalproex Combination Versus Divalproex Monotherapy in the Treatment of Bipolar Mixed Episodes: A Double-Blind, Placebo-Controlled Study. J Clin Psychiatry: 70(11) 1540-7.
22. Kane JM, Correll CU, Goff DC, Kirkpatrick B, Marder SR et al. (2009) A Multicenter, Randomized, Double-Blind, Placebo-Controlled, 16-Week Study of Adjunctive Aripiprazole for Schizophrenia or Schizoaffective Disorder Inadequately Treated With Quetiapine or Risperidone Monotherapy. J Clin Psychiatry: 70(10) 1348-57.
23. Kane JM, D’Souza DC, Patkar AA, Youakim JM, Tiller JM et al. (2010) Armodafinil as Adjunctive Therapy in Adults With Cognitive Deficits Associated With Schizophrenia: A 4-Week, Double-Blind, Placebo-Controlled Study. J Clin Psychiatry: 71(11) 1475-81.
24. Kane JM, Sanchez R, Perry PP, Jin N, Johnson BR et al. (2012) Aripiprazole intramuscular depot as maintenance treatment in patients with schizophrenia: a 52-week, multicenter, randomized, double-blind, placebo-controlled study. J Clin Psychiatry: 73(5) 617-24.
25. Katon W, Russo J, Lin EHB, Schmittdiel J, Ciechanowski P et al. (2012) Cost-effectiveness of a Multicondition Collaborative Care Intervention: A Randomized Controlled Trial. Arch Gen Psychiatry: 69(5) 506-14.
26. Kim S, Wollburg E and Roth WT (2012) Opposing Breathing Therapies for Panic Disorder: A Randomized Controlled Trial of Lowering vs Raising End-Tidal Pco2. J Clin Psychiatry: 73(7) 931-9.
27. Kornstein SG, Jiang Q, Reddy S, Musgnung JJ and Guico-Pabia CJ (2010) Short-Term Efficacy and Safety of Desvenlafaxine in a Randomized, Placebo-Controlled Study of Perimenopausal and Postmenopausal Women With Major Depressive Disorder. J Clin Psychiatry: 71(8) 1088-96.
28. Krystal AD, Harsh JR, Yang R, Rippon GA, Lankford DA (2010) A Double-Blind, Placebo-Controlled Study of Armodafinil for Excessive Sleepiness in Patients With Treated Obstructive Sleep Apnea and Comorbid Depression. J Clin Psychiatry: 71(1) 32-40.
29. Lespérance F, Frasure-Smith N, St-André E, Turecki G, Lespérance P et al. (2011) The Efficacy of Omega-3 Supplementation for Major Depression: A Randomized Controlled Trial. J Clin Psychiatry: 72(8) 1054-62.
30. Lyoo IK, Yoon S, Kim T, Hwang J, Kim JE et al. (2012) A Randomized, Double-Blind Placebo-Controlled Trial of Oral Creatine Monohydrate Augmentation for Enhanced Response to a Selective Serotonin Reuptake Inhibitor in Women With Major Depressive Disorder. Am J Psychiatry: 169(9) 937-45.
31. Manor I, Ben-Hayun R, Aharon-Peretz J, Salomy D, Weizman A et al. (2012) A Randomized, Double-Blind, Placebo-Controlled, Multicenter Study Evaluating the Efficacy, Safety, and Tolerability of Extended-Release Metadoxine in Adults With Attention-Deficit/Hyperactivity Disorder. J Clin Psychiatry: 73(12) 1517-23.
32. Manor I, Ben-Hayun R, Aharon-Peretz J, Salomy D, Weizman A et al. (2010) Adjunctive Armodafinil for Major Depressive Episodes Associated With Bipolar I Disorder: A Randomized, Multicenter, Double-Blind, Placebo-Controlled, Proof-of-Concept Study. J Clin Psychiatry: 71(10) 1363-70.
33. Mariani JJ, Pavlicova M, Bisaga A, Nunes EV, Brooks DJ et al. (2012) Extended-Release Mixed Amphetamine Salts and Topiramate for Cocaine Dependence: A Randomized Controlled Trial. Biol Psychiatry: 72(11) 950-6.
34. McEvoy JP, Citrome L, Hernandez D, Cucchiaro J, Hsu J et al. (2013) Effectiveness of Lurasidone in Patients with Schizophrenia or Schizoaffective Disorder Switched From Other Antipsychotics: A Randomized, 6-Week, Open-Label Study. J Clin Psychiatry: 74(2) 170-9.
35. Mischoulon D, Witte J, Levy M, Papakostas GI, Pet LR et al. (2012) Efficacy of Dose Increase Among Nonresponders to Low-Dose Aripiprazole Augmentation in Patients With Inadequate Response to Antidepressant Treatment: A Randomized, Double-Blind, Placebo-Controlled, Efficacy Trial. J Clin Psychiatry: 73(3) 353-7.
36. Morland LA, Greene CJ, Rosen CS, Foy D, Reilly P et al. (2010) Telemedicine for Anger Management Therapy in a Rural Population of Combat Veterans With Posttraumatic Stress Disorder: A Randomized Noninferiority Trial. J Clin Psychiatry: 71(7) 855-63.
37. Nations KR, Smits JAJ, Tolin DF, Rothbaum BO, Hofmann SG et al. (2012) Evaluation of the Glycine Transporter Inhibitor Org 25935 as Augmentation to Cognitive-Behavioral Therapy for Panic Disorder: A Multicenter, Randomized, Double-Blind, Placebo-Controlled Trial. J Clin Psychiatry: 73(5) 647-53.
38. Nierenberg AA, Friedman ES, Bowden CL, Sylvia LG, Thase ME et al. (2013) Lithium Treatment Moderate-Dose Use Study (LiTMUS) for Bipolar Disorder: A Randomized Comparative Effectiveness Trial of Optimized Personalized Treatment With and Without Lithium. Am J Psychiatry: 170(1) 102-10.
39. Papakostas GI, Vitolo OV, IsHak WW, Rapaport MH, Zajecka JM et al. (2012) A 12-Week, Randomized, Double-Blind, Placebo-Controlled, Sequential Parallel Comparison Trial of Ziprasidone as Monotherapy for Major Depressive Disorder. J Clin Psychiatry: 73(12) 1541-7.
40. Philip NS, Carpenter LL, Tyrka AR, Whiteley LB and Price LH (2009) Varenicline Augmentation in Depressed Smokers: An 8-Week, Open-Label Study. J Clin Psychiatry: 70(7) 1026-31.
41. Pollack MH, Hoge EA, Worthington JJ, Moshier SJ, Wechsler RS et al. (2011) Eszopiclone for the Treatment of Posttraumatic Stress Disorder and Associated Insomnia: A Randomized, Double-Blind, Placebo-Controlled Trial. J Clin Psychiatry: 72(7) 892-7.
42. Rickels K, Athanasiou M, RobinsonDS, Gibertini M, Whalen H et al. (2009) Evidence for Efficacy and Tolerability of Vilazodone in the Treatment of Major Depressive Disorder: A Randomized, Double-Blind, Placebo-Controlled Trial. J Clin Psychiatry: 70(3) 326-33.
43. Ritsner MS, Miodownik C, Ratner Y, Shleifer T, Mar M et al. (2011) L-Theanine Relieves Positive, Activation, and Anxiety Symptoms in Patients With Schizophrenia and Schizoaffective Disorder: An 8-Week, Randomized, Double-Blind, Placebo-Controlled, 2-Center Study. J Clin Psychiatry: 72(1) 34-42.
44. Ritterband LM, Thorndike FP, Gonder-Frederick LA, Magee JC, Bailey ET et al. (2009) Efficacy of an Internet-Based Behavioral Intervention for Adults With Insomnia. Arch Gen Psychiatry: 66(7) 692-8.
45. Rosenthal JZ, Boyer P, Vialet C, Hwang E and Tourian, KA. (2013) Efficacy and Safety of Desvenlafaxine 50 mg/d for Prevention of Relapse in Major Depressive Disorder: A Randomized Controlled Trial. J Clin Psychiatry: 74(2) 158-66.
46. Rossouw TI and Fonagy P (2012) Mentalization-Based Treatment for Self-Harm in Adolescents: A Randomized Controlled Trial. J Am Acad Child Adolesc Psychiatry:51(12) 1304-1313.
47. Sachs GS, Ice KS, Chappell PB, Schwartz JH, Gurtovaya O et al. (2011) Efficacy and Safety of Adjunctive Oral Ziprasidone for Acute Treatment of Depression in Patients With Bipolar I Disorder: A Randomized, Double-Blind, Placebo-Controlled Trial. J Clin Psychiatry: 72(10) 1413-22.
48. Sachs GS, Vanderburg DG, Karayal ON, Kolluri S, Bachinsky M et al. (2012) Adjunctive Oral Ziprasidone in Patients With Acute Mania Treated With Lithium or Divalproex, Part 1: Results of a Randomized, Double-Blind, Placebo-Controlled Trial. J Clin Psychiatry: 73(11) 1412-9.
49. Schuppert HM, Timmerman ME, Bloo J, van Gemert TG, Wiersema HM et al. (2012) Emotion Regulation Training for Adolescents With Borderline Personality Disorder Traits: A Randomized Controlled Trial. J Am Acad Child Adolesc Psychiatry: 51(12) 1314-1323.
50. Singh J, Robb A, Vijapurkar U, Nuamah I, and Hough D. (2011) A Randomized, Double-Blind Study of Paliperidone Extended-Release in Treatment of Acute Schizophrenia in Adolescents. Biol Psychiatry: 70(12) 1179-87.
51. Somoza EC, Winship D, Gorodetzky CW, Lewis D, Ciraulo DA et al. (2013) A Multisite, Double-blind, Placebo-Controlled Clinical Trial to Evaluate the Safety and Efficacy of Vigabatrin for Treating Cocaine Dependence. JAMA Psychiatry: 70(6) 630-7.
52. Stahl SM, Cucchiaro J, Simonelli D, Hsu J, Pikalov A et al. (2013) Effectiveness of Lurasidone for Patients With Schizophrenia Following 6 Weeks of Acute Treatment With Lurasidone, Olanzapine, or Placebo: A 6-Month, Open-Label, Extension Study. J Clin Psychiatry: 74(5) 507-15.
53. Stahl SM, Fava M, Trivedi MH, Caputo A, Shah A et al. (2010) Agomelatine in the Treatment of Major Depressive Disorder: An 8-Week, Multicenter, Randomized, Placebo-Controlled Trial. J Clin Psychiatry: 71(5) 616-26.
54. Stein DJ, Ahokas A, Albarran C, Olivier V, Allgulander C (2012) Agomelatine Prevents Relapse in Generalized Anxiety Disorder: A 6-Month Randomized, Double-Blind, Placebo-Controlled Discontinuation Study. J Clin Psychiatry: 73(7) 1002-8.
55. Stroup TS, McEvoy JP, Ring KD, Hamer RH, Vange LM et al. (2011) A randomized Trial examining the effectiveness of Switching From olanzapine, Quetiapine, or risperidone to Aripiprazole to reduce Metabolic risk : Comparison of Antipsychotics for Metabolic Problems (CAMP). Am J Psychiatry: 168(9) 947-56.
56. Tariot PN, Schneider LS, Cummings J, Thomas RG, Raman R et al. (2011) Chronic Divalproex Sodium to Attenuate Agitation and Clinical Progression of Alzheimer Disease. Arch Gen Psychiatry: 68(8) 853-61.
57. Tiihonen J, Krupitsky E, Verbitskaya E, Blokhina E, Mamontova O et al. (2012) Naltrexone Implant for the Treatment of Polydrug Dependence: A Randomized Controlled Trial. Am J Psychiatry: 169(5) 531-6.
58. Watanabe N, Furukawa TA, Shimodera S, Morokuma I, Katsuki F et al. (2011) Brief Behavioral Therapy for Refractory Insomnia in Residual Depression: An Assessor-Blind, Randomized Controlled Trial. J Clin Psychiatry: 72(12) 1651-8.
59. Weiss RD, Potter JS, Fiellin DA, Byrne M, Connery HS et al. (2011) Adjunctive Counseling During Brief and Extended Buprenorphine-Naloxone Treatment for Prescription Opioid Dependence: A 2-Phase Randomized Controlled Trial. Arch Gen Psychiatry: 68(12)1238-46.
60. Winhusen TM, Somoza EC, Brigham GS, Liu DS, Green CA et al. (2010) Impact of Attention-Deficit/Hyperactivity Disorder (ADHD) Treatment on Smoking Cessation Intervention in ADHD Smokers: A Randomized, Double-Blind, Placebo-Controlled Trial. J Clin Psychiatry: 71(12) 1680-8.
